# Selection Pressure in Alternative Reading Frames

**DOI:** 10.1371/journal.pone.0108768

**Published:** 2014-10-01

**Authors:** Katharina Mir, Steffen Schober

**Affiliations:** Institute of Communications Engineering, Ulm University, Ulm, Germany; University of Illinois, United States of America

## Abstract

Overlapping genes are two protein-coding sequences sharing a significant part of the same DNA locus in different reading frames. Although in recent times an increasing number of examples have been found in bacteria the underlying mechanisms of their evolution are unknown. In this work we explore how selective pressure in a protein-coding sequence influences its overlapping genes in alternative reading frames. We model evolution using a time-continuous Markov process and derive the corresponding model for the remaining frames to quantify selection pressure and genetic noise. Our findings lead to the presumption that, once information is embedded in the reverse reading frame −2 (relative to the mother gene in +1) purifying selection in the protein-coding reading frame automatically protects the sequences in both frames. We also found that this coincides with the fact that the genetic noise measured using the conditional entropy is minimal in frame −2 under selection in the coding frame.

## Introduction

Overlapping genes are protein coding genes sharing the same DNA locus in different reading frames. As DNA consists of two strands and each amino acid is encoded by non-overlapping triplets (codons), up to six reading frames are possible at a given locus. Overlapping genes are a well known and accepted phenomenon in viruses, however this effect was explained from space limitations of the capsid volume [1]. Until lately most authors denied the existence of overlapping genes in bacterial genomes, consequently bacterial genome annotation programs excluded overlapping candidates in alternative reading frames deliberately [2]–[5]. Although an experimental verification of two protein-coding genes in the same DNA locus is extremely challenging, over the last years an increasing number of non trivially overlapping genes in prokayotes have been found [6]–[10].

This paper is concerned with the question how selection pressure in the protein-coding frame influences alternative reading frames. Is it possible to protect by selection two protein-coding sequences simultaneously? We explore this question using a stochastic model for the evolution of the protein-coding reading frame and predict the consequent behaviour in the alternative reading frames.

Sequence evolution can be described on nucleotide level [11]–[14], amino acid level, e.g. Dayhoff and Schwartz [15], or on codon level. Here we chose the latter approach using a time-continuous Markov process as suggested by Goldman and Yang [16] and Muse and Gaut [17]. We apply the model of Yang and Nielsen [18] which is based on [16]. An extended model was already used by Sabath *et al.* [19] to study the evolution of a random protein-coding sequence. In contrast to our approach, Sabath investigated the selection intensities of overlapping genes assuming that each gene of the overlapping pair faces selection independently.

Several studies analyzed selection intensities in virus genomes within overlapping gene regions investigating how nonsynonymous and synonymous mutations influence two reading frames simultaneously showing that a high rate of nonsynonymous mutations in one reading frame falls onto synonymous substitutions in an alternative frame at the same time, e.g. [20]–[22].

Our investigation reveals that selection pressure in the protein-coding reading frame +1 is correlated to the reverse reading frame −2, where in fact many examples of overlapping genes found so far are located e.g., [9], [10]. Precisely there is a strong coupling of the nonsynonymous to synonymous substitutions rate ratios in these frames. In another approach following Yockey [23], we quantify the genetic noise using the conditional entropy and the mutual information as a measure of sequence similarity. The results obtained coincides with the former observations.

The outline of the paper is as follows: In Section *Methods* we introduce the evolutionary framework and the calculation of selection pressure. The biological and information theoretic measures are presented in Section *Results*, together with an application of the model to a bacterial genome and evidence on the robustness of our approach. Finally we discuss the results in the last section.

## Methods

### Framework of Evolutionary Model

This section introduces the evolutionary framework and the notations used. We denote a discrete random variable with *X* and their corresponding probability mass function with 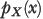, where *x* is the concrete realization of *X*. Throughout the paper the nucleotide alphabet is denoted with 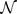 = {*A*, *C*, *G*, *T*} and the codon alphabet is denoted with 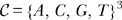.

In the following we consider the well known Goldman and Yang model [16] in a simplified version as it was introduced by [18], where the following definitions can be found. (For more details on the derivation of the model see [24].) The model assumes a stationary codon distribution and independence of the evolving codon sites. The evolution of protein-coding DNA sequences is modelled by a time-continous Markov process described by the substitution rate matrix *Q* = {*q_xy_*}, where *q_xy_* is the rate from codon *x* to codon *y* with *x*≠*y*

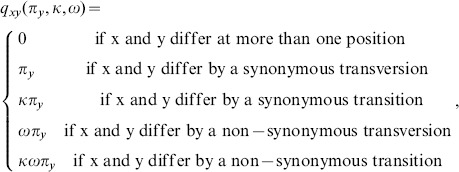
(1)where *κ* is the transition/transversion rate, *ω* is the nonsynonymous/synonymous rate ratio and *π_y_* is the equilibrium frequency of codon *y*. Note that 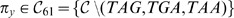 as transitions to stop codons are not allowed inside functional proteins. The row sums of the rate matrix *Q* = {*q_xy_*} have to be zero, which determines the main diagonal of the matrix. Further the rate matrix is multiplied by a scaling factor to normalize the expected number of nucleotide substitutions per codon to one. With every time-continuous Markov process, a discrete time Markov chain can be associated. This leads to a discrete evolution matrix 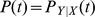 with conditional probabilities that describes a transition of an input 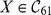 to an output 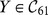 for a fixed *t*. The evolutionary transition probability matrix is determined by 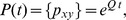
where *p_xy_* is the probability that input codon *x* becomes *y* after time t. Note that
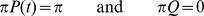
holds, where row vector *π* is the stationary codon distribution.

We call 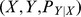 an evolutionary channel referring to the communication theoretic term [25]. Note that the rate matrix *Q* is also a channel matrix. Further the parameters of the rate matrix *t*, *ω* and *κ* are arbitrary but fixed.

**Figure 1:**
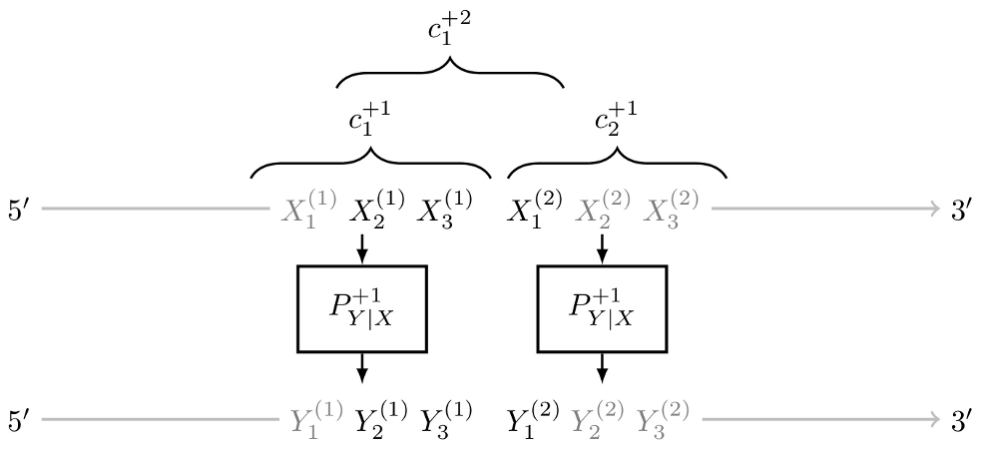
Transition Scheme. Scheme of transitions in sequence direction on forward strand and in time direction.

Given a rate matrix for the protein-coding reading frame, we are interested in computing the resulting rate and evolutionary channel matrices in the other reading frames. We define the protein-coding reading frame as +1 and denote the shifted and reverse complement reading frames as non-coding reading frames *f* = {−1, ±2, ±3}. If we refer to a special reading frame, we use the index *f*. The setup we consider is as follows: In the protein-coding reading frame we assume that codons 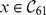 with codon usage *π* ^+1^ from a bacterial organism are transmitted independently over the evolutionary channel *P*(*t*, *κ*, *ω*, *π*) to the output *y*. This is called a discrete memoryless channel. For convenience we write 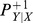 instead of 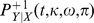. Each codon in frame +1 consists of three random variables 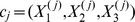 with realizations 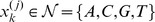 and evolves to codon 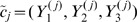 with 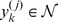. In frame +1 we observe the scheme presented in Figure 1. Given 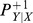 and *π* ^+1^ we want to determine the evolution matrix per reading frame 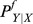, 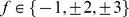. We solve this task directly via the rate matrix per reading frame *Q^f^* given the rate matrix *Q* ^+1^ and *π* ^+1^ such that we are independent of the evolution time. For the alternative reading frames we combine two independent time-continuous Markov chains to the corresponding di-codon matrix in frame +1 by
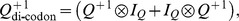
where ⊗ is the Kronecker product and *I_Q_* is the identity matrix with the same dimension as the rate matrix [26]. The rates of the di-codon transitions are now combined to compute the rate matrices in the other frames. Without loss a generality, we consider frame +2 (black parts in Figure 1).
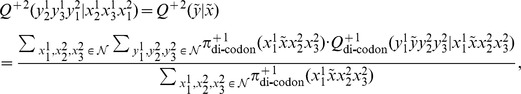
where 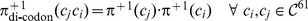. The rate matrices of the other frames can be determined accordingly. Note that *Q^f^* is a 64×64 matrix for *f* = {±2, ±3} and a 61×61 matrix for *f* = {±1}. Given the rate matrix *Q^f^* of a time-continuous Markov chain the corresponding stationary distribution π*^f^* in each reading frame as well as the transition matrix 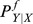 for time *t* can be easily determined.

### Selection pressure during evolution

An important parameter describing the selection pressure on the protein level is the ratio of nonsynonymous *d_N_* to synonymous *d_S_* substitution rates, denoted with 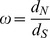, see e.g., [16]. Three basic scenarios are distinguished e.g., [27]: Purifying selection when *ω*<1, adaptive selection for *ω*>1 and neutral mutation if *ω* = 1. To determine the nonsynonymous/synonymous rate ratio *ω* in each reading frame, we apply the procedure presented in [18] and [24], that is based on the transition probability matrix 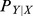, but can be easily adapted to the rate matrix *Q*.

Assume we determined, the rate matrix *Q^f^* in each frame 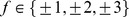 as presented in *Framework of Evolutionary Model* as well as the stationary distributions *π^f^*. The proportion of synonymous substitutions is the sum over all codon pairs *x* and *y* (*x*≠*y*) that code for the same amino acid 
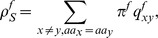
where *aa_x_* is the amino acid encoded by codon *x*. The proportion of nonsynonymous substitutions is calculated accordingly by 
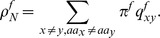



The transition/tranversion rate 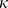 is the same in all reading frames. We assume that it is known from reading frame +1. To determine the proportion of synonymous sites, we calculate a new rate matrix following Eq. (1) for a fixed *ω* = 1.0, 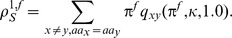



The proportion of nonsynonymous sites is calculated accordingly and denoted with 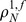. The number of synonymous substitutions per synonymous site is 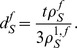



The number of nonsynonymous substitutions per nonsynonymous site 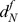 is calculated accordingly. This results in 
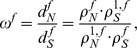
where the time as well as scaling factors of the rate matrices cancel out.

## Results

Throughout the paper, we use the model genome *Escherichia coli* O157:H7 EDL933 (Accession number NC_002655, abbreviation EHEC), with a GC content of 50.4% and a length of 5528445 base pairs. In *File S1 Model verification* we present some simulation results to validate the calculations of the equilibrium frequencies per reading frame π*^f^*.

**Figure 2:**
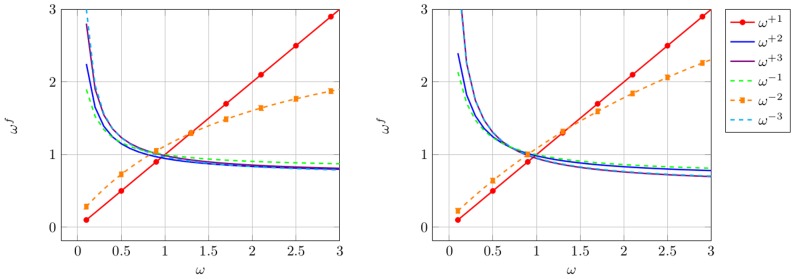
Selection Pressure. Estimation of nonsynonymous/synonymous rate ratio *ω* ^*f*^ for different parameter settings. In the left panel *κ* = 1.0, *t* = 1.0 and on the right panel we set *κ* = 5.0, *t* = 1.0. Protection of protein-coding frame +1 for *ω*<1 is directly coupled with a protection of reading frame −2.

To investigate the influence of selection pressure during evolution we chose two different input scenarios: The transition/transversion rates are *κ* = 1.0 or *κ* = 5.0 at time *t* = 1.0 and the nonsynonymous/synonymous rate ratio *ω* takes values between [0, 3]. The calculation of the nonsynonymous/synonymous rate ratio *ω^f^* for 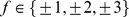 reveals the following results. Purifying selection refers to a selection against nonsynonymous substitutions on the DNA level, which protects the sequence. In Figure 2, we see that a protection of the coding frame +1 with *ω*<1, also protects the sequence in frame −2, the other alternative frames face adaptive selection. The opposite is observed for *ω*>1, where new information can be induced in frames +1 and −2, whereas the other frames are slightly below the neutral mutation line. The behaviour of *ω^f^* is consistent for both scenarios. Note, there are numerous methods to determine the synonymous to nonsynonymous rate ratio. The *File S1 Selection pressure* shows a comparison of our approach with an alternative method.

### Quantifying noise during evolution

In this part, we deal with the following question: An amino acid is transmitted over the evolutionary channel, how long is this information conserved in the different reading frames?

Evolution of a sequence can be considered as a communication process over time. In his book [23] proposed to use the conditional entropy to measure the amount of genetic information that can be transmitted over a noisy channel (based on [25]). We define the amino acid alphabet 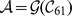, where 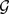 is the genetic code, which results in a cardinality of 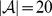. The codon evolution matrix per frame 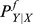 can be summarized to determine the amino acid evolution matrix 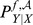, based on the stationary distribution *π^f^*. The stop codon probabilities are removed in all frames. The conditional entropy between two random variables *X* and *Y* over alphabets 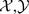 is defined as, e.g., in [28]: 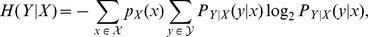
where 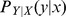 is the conditional probability.

The conditional entropy between two randomly chosen amino acids *X* and *Y* in frame *f* conditioned on *X* = a with 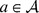 is accordingly 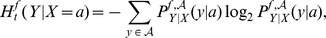
where 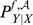 is the amino acid substitution matrix per reading frame. If we know that a specific amino acid was transmitted, how much of this knowledge is lost after time 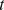
? As the comparison of 20 values over time is inconvenient, we apply uniform weighting according to the amino acids 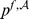, which results in 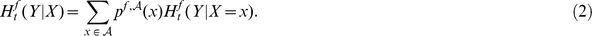
Note that Eq. (2) is bounded by 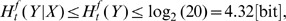
where the entropy (or uncertainty) 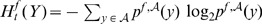 is maximal for a uniformly distributed random variable *Y*.

Yockey [23] additionally suggests the application of the mutual information as a measure of similarity between sequences. The mutual information between amino acid *X* and *Y* per frame *f* is defined as, e.g., in [28]: 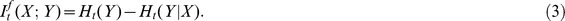
Note, that the channel capacity, which is the maximal mutual information for all input distributions, can be determined numerically using the Blahut-Arimoto algorithm. But as there is no direct interpretation in our framework and the results match those of the mutual information, we abandoned the presentation.

#### Results at the example of EHEC

**Figure 3:**
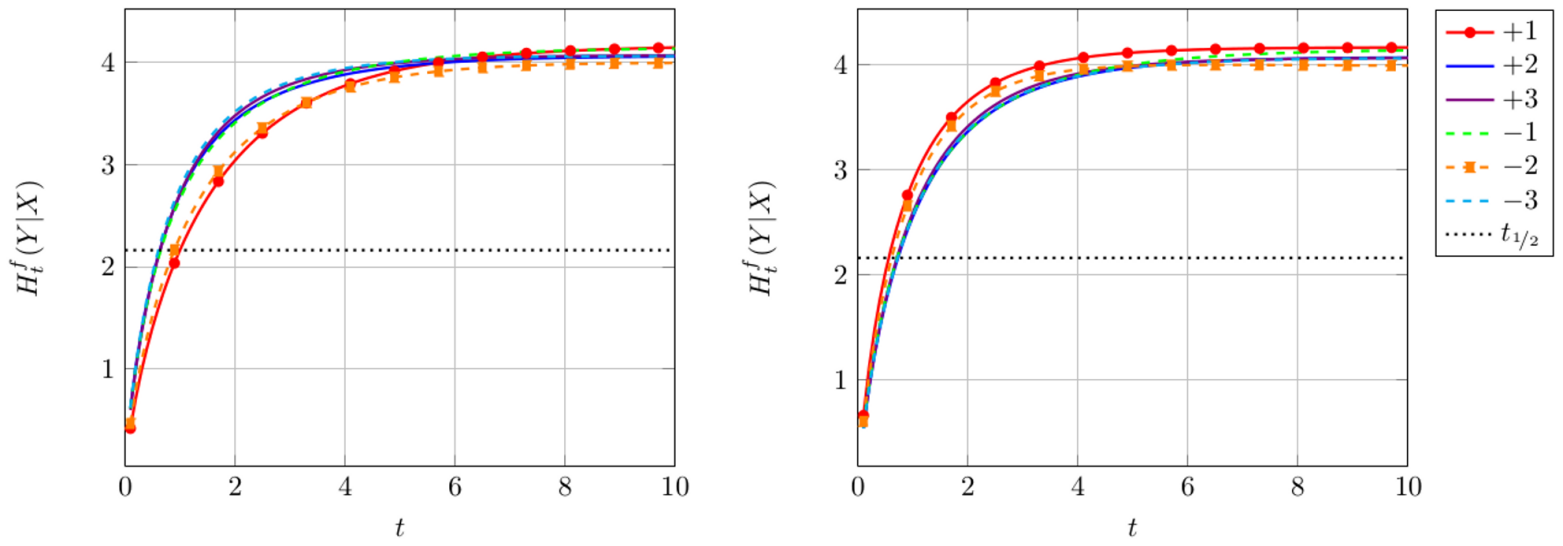
Information Loss. Conditional entropy for uniform input distribution over amino acids for different values of *ω* and *κ* = 1.0. On the left *ω* = 0.3, the protein-coding frame as well as frame −2 are protected, which results in a slower information loss than for the other reading frames. On the right *ω* = 3.0, we see the opposite scenario. At the black dotted line, half of the information is lost.

We chose two different input scenarios: Set *ω* = 0.3 to model purifying selection and *ω* = 3.0 to model adaptive selection. The transition/transversion rate is fixed to *κ* = 1.0 and the time *t* is changed during simulation. The same parameter setting was already used in [27]. We apply the conditional entropy introduced in Eq. (2) to answer the question how long the information which amino acid was transmitted is conserved in the different reading frames. The results are presented in Figure 3.

Evolution means loss of information over time or from a complementary point of view, an increase of uncertainty. To quantify this information loss, we determine the time needed to loose half of the information. As the conditional entropy in bounded by log_2_(20), we determine for each frame 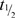 such that 
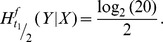



The results are summarized in Table 1.

**Table 1:** Time for each frame where the conditional entropy is 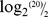.

*ω*	+1	+2	+3	−1	−2	−3
0.3	1.0	0.7	0.7	0.7	0.9	0.7
3.0	0.6	0.8	0.8	0.8	0.7	0.8

In accordance with the results of *ω^f^* in Figure 2 we interpret Figure 3 and Table 1 as follows. Protected frames with *ω*<1, store information longer than the unprotected frames with *ω*>1. When frame +1 is protected, then the −2 frame is protected automatically, therefore those frames show a slower increasing uncertainty than the alternative frames.

**Figure 4:**
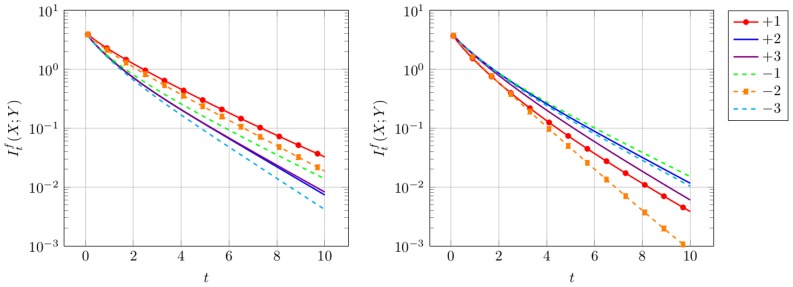
Sequence Similarity. Mutual information for uniform input distribution over amino acids for different values of *ω* and *κ* = 1.0. On the left *ω* = 0.3, the amount of information transmitted over the channel is largest for the protected frames +1 and −2. On the right *ω* = 3.0, where those frames are not protected, the opposite holds.

Now, the mutual information 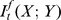 per reading frame 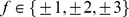 is investigated applying Eq. (3). The mutual information measures the similarity between *X* and *Y*, which is directly connected to the amount of information that can be transmitted over the channel [23]. We observe for the first scenario, where *ω* = 0.3, presented in the left panel of Figure 4 that most information can be transmitted in reading frame +1 followed by reading frame −2. In general the proportion of information, that can be transmitted over the evolutionary channel decreases over time, but this information loss is faster in the frames, where *ω^f^*>1. In the right panel of Figure 4, where *ω* = 3.0, we see that the mutual information is smallest, for the frames +1 and −2 which is also in accordance with Figure 2. This observation is confirmed in the *File S1 Conditional entropy and mutual information* for different values of *ω*.

#### Robustness of method

**Figure 5:**
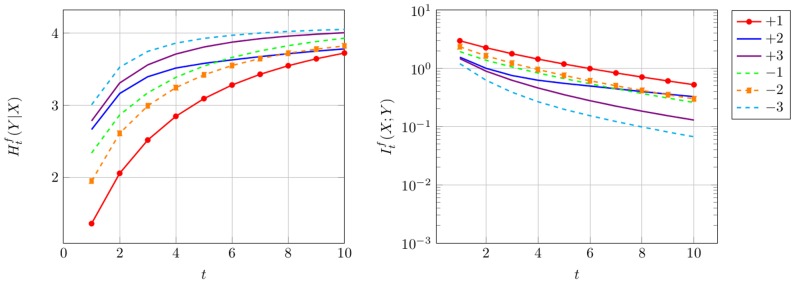
Empirical Substitution Matrix. Estimation of conditional entropy (left panel) and mutual information (right panel) for empirical codon substitution matrix *P_ECM_*. A slower information loss for reading frames −2 is observed due to a protection of the protein-coding reading frame +1.

The question arises, how robust our method is, if we choose another codon substitution matrix. As we are able to determine the mutual information and the conditional entropy per reading frame, given only the evolution matrix in the coding reading frame 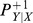 and the stationary distribution of EHEC *π* ^+1^ it is also possible to substitute the channel matrix 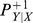. In 2005 [29] published an empirical codon substitution matrix (*P_ECM_*) obtained from an alignment of vertebrate DNA, which can also be applied to bacteria. Given a transition matrix we present in *File S1 Robustness of results* a method to estimate the transition matrices per reading frame based on the channel matrix 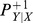. For our investigations, the different time points *t* presented in Figure 5 are obtained by 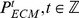. The results confirm our findings, that most information can be transmitted in +1, followed by −2. That makes sense, as the matrix is based on genes with purifying selection, otherwise they would not have survived over time.

## Summary

In this paper we introduced a model to determine the codon evolution in different reading frames based on the protein-coding reading frame +1. The model is used to predict the selection pressure within different reading frames and reveals that a protection of the protein-coding reading frame also preserves the reverse reading frame −2. For the case of adaptive selection both frames are free to evolve. The remaining alternative frames show the reverse relation, i.e. they give preference to nonsynonymous substitutions while reading frame +1 is protected and are preserved when +1 is exposed to adaptive selection. These findings are further confirmed by the presented results on the conditional entropy. Namely, if *ω*<1, the genetic noise is minimal in frames +1 and −2, also the sequence similarity measured by the mutual information is largest. Conversely for *ω*>1, where the genetic noise of +1 and −2 is largest and the sequence similarity accordingly smallest.

## Discussion and Conclusion

At a first glance, understanding the evolution of overlapping protein-coding regions is extremely challenging, because one DNA segment codes for two proteins which are translated in different reading frames simultaneously, such that a mutation affects both proteins [30], [31]. Biologist investigate evolutionary adaption of proteins for years now, assuming that adaption requires more nucleotide mutations at positions that change an amino acid than at positions that preserve a site [32]. The parameter of choice that measures the substitution rate at those sites is 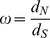 and is therefore used as an indicator of selective pressure within genes.

Meanwhile a large field emerged, investigating the evolutionary constraints within overlapping and non overlapping reading frames [30], [33]–[36]. There exist empirical analyses describing, that a loss of a stop codon within a protein-coding gene by deletion, mutation or frame-shift, causes an elongation to the next stop codon, whereby an overlapping pair originates [37], [38]. Other studies suggest, that the loss of a start codon is responsible for the development of an overlap [39]–[41]. From this point of view, a random formation can not be ruled out.

Our point of interest is slightly different, assuming we are given a protein-coding reading frame that evolves over time, we are interested in the evolutionary constraints implied within alternative reading frames. A biological interpretation of our findings is that during adaption many mutations occur that change amino acids in reading frames +1 and −2 simultaneously. Once a protein in reading frame +1 is fixed and adaptive selection is replaced by purifying selection, this process stops and the amount of synonymous substitutions increases, again in both reading frames. Note that we make no statement that both reading frames are already translated into proteins, since function of a sequence could also evolve later. As a matter of fact, over time the divergence of a sequence always increases even if it is *protected*, but we showed that this change happens slower in case of purifying selection for both, the +1 and −2 reading frame. No matter, how or if an overlapping gene pair evolved, our observations indicate the special role of the −2 reading frame. Interestingly, two recently experimentally verified examples of overlapping gene pairs in bacteria *yaaW/htga* by [10] and *dmdR1/adm* by [9] are in frame −2. We showed that it is possible to protect this frame by simply controlling the selection pressure within the protein-coding reading frame. This can be attributed to a property of the genetic code, as the most important codon positions are the first and second which fall onto the second respectively first position in the −2 frame. But this could also mean that a conserved sequence in −2 might be solely an artefact, providing not necessarily evidence for functionality.

Finally note that it is challenging to embed information in the overlapping reading frame −2, when the protein-coding reading frame +1 has a fixed amino acid sequence. Assume two amino acids 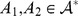, where 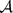 ^*^ is 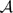 plus a stop label, should be encoded in the coding reading frame. Obviously each amino acid corresponds to an individual number of codons, hence it is possible to encode a certain number of different amino acids in the alternative reading frames without changing *A*_1_ and *A*_2_. The average taken over all possible pairs *A*_1_, *A*_2_ are shown in Table 2; it turns out that the degree of freedom is smallest in −2. It is worth noting that in general it is possible to embed information even in protein-coding sequences, see for example [42].

**Table 2:** Degree of freedom to choose amino acids according to the genetic code.

Frame	+2	+3	−1	−2	−3
Mean	2.94	2.93	2.67	1.59	3.12

## Supporting Information

File S1 **Additional Data and Figures.** Contains further information to verify the model predictions by comparison with simulation, another method to determine the selection pressure, different investigations on the conditional entropy and mutual information as well as a method to show the robustness of results.
(PDF)

## References

[1] Chirico N, Vianelli A, Belshaw R (2010) Why genes overlap in viruses. Proceedings of the Royal Society B: Biological Sciences 277: 3809–3817. 20610432 10.1098/rspb.2010.1052 PMC2992710

[2] Yooseph S, Sutton G, Rusch DB, Halpern AL, Williamson SJ, et al (2007) The Sorcerer II Global Ocean Sampling expedition: expanding the universe of protein families. PLoS Biology 5: e16+. 17355171 10.1371/journal.pbio.0050016 PMC1821046

[3] Delcher AL, Bratke KA, Powers EC, Salzberg SL (2007) Identifying bacterial genes and endosymbiont DNA with Glimmer. Bioinformatics 23: 673–679. 17237039 10.1093/bioinformatics/btm009 PMC2387122

[4] Warren A, Archuleta J, Feng WC, Setubal J (2010) Missing genes in the annotation of prokaryotic genomes. BMC Bioinformatics 11: 131. 20230630 10.1186/1471-2105-11-131 PMC3098052

[5] Johnson ZI, Chisholm SW (2004) Properties of overlapping genes are conserved across microbial genomes. Genome Research 14: 2268–2272. 15520290 10.1101/gr.2433104 PMC525685

[6] McVeigh A, Fasano A, Scott D, Jelacic S, Moseley S, et al (2000) IS1414, an *Escherichia coli* insertion sequence with a heat-stable enterotoxin gene embedded in a transposase-like gene. Infection and Immunity 68: 5710–5715. 10992475 10.1128/iai.68.10.5710-5715.2000 PMC101527

[7] Behrens M, Sheikh J, Nataro JP (2002) Regulation of the overlapping *pic/set* locus in *Shigella flexneri* and enteroaggregative *Escherichia coli* . Infection and Immunity 70: 2915–2925. 12010980 10.1128/IAI.70.6.2915-2925.2002 PMC127977

[8] Silby MW, Levy SB (2008) Overlapping protein-encoding genes in *Pseudomonas fluorescens* Pf0-1. PLoS Genetics 4: e1000094. 18551168 10.1371/journal.pgen.1000094 PMC2396522

[9] Tunca S, Barreiro C, Coque JJR, Martin JF (2009) Two overlapping antiparallel genes encoding the iron regulator DmdR1 and the Adm proteins control siderophore and antibiotic biosynthesis in *Streptomyces coelicolor* A3(2). FEBS Journal 276: 4814–4827. 19664059 10.1111/j.1742-4658.2009.07182.x

[10] Fellner L, Bechtel N, Witting MA, Simon S, Schmitt-Kopplin P, et al (2013) Phenotype of htga (mbia), a recently evolved orphan gene of escherichia coli and shigella, completely overlapping in antisense to yaaw. FEMS Microbiology Letters: 1–8. 10.1111/1574-6968.12288 24111745

[11] Jukes TH, Cantor CR (1969) Evolution of Protein Molecules. Academy Press.

[12] Kimura M (1980) A simple method for estimating evolutionary rates of base substitutions through comparative studies of nucleotide sequences. Journal of Molecular Evolution 16: 111–120. 7463489 10.1007/BF01731581

[13] Felsenstein J (1981) Evolutionary trees from dna sequences: a maximum likelihood approach. Journal of Molecular Evolution 17: 368–376. 7288891 10.1007/BF01734359

[14] Hasegawa M, Kishino H, aki Yano T (1985) Dating of the human-ape splitting by a molecular clock of mitochondrial dna. Journal of Molecular Evolution 22: 160–174. 3934395 10.1007/BF02101694

[15] Dayhoff MO, Schwartz RM (1978) Chapter 22: A model of evolutionary change in proteins. In: in Atlas of Protein Sequence and Structure.

[16] Goldman N, Yang Z (1994) A codon-based model of nucleotide substitution for protein-coding DNA sequences. Molecular Biology and Evolution 11: 725–736. 7968486 10.1093/oxfordjournals.molbev.a040153

[17] Muse SV, Gaut BS (1994) A likelihood approach for comparing synonymous and nonsynonymous nucleotide substitution rates, with application to the chloroplast genome. Molecular Biology and Evolution 11: 715–724. 7968485 10.1093/oxfordjournals.molbev.a040152

[18] Yang Z, Nielsen R (2000) Estimating synonymous and nonsynonymous substitution rates under realistic evolutionary models. Molecular Biology and Evolution 17: 32–43. 10666704 10.1093/oxfordjournals.molbev.a026236

[19] Sabath N, Landan G, Graur D (2008) A method for the simultaneous estimation of selection intensities in overlapping genes. PLoS One 3. 10.1371/journal.pone.0003996 PMC2601044 19098983

[20] Guyader S, Ducray D (2002) Sequence analysis of Potato leafroll virus isolates reveals genetic stability, major evolutionary events and differential selection pressure between overlapping reading frame products. Journal of General Virology 83: 1799–807. 12075102 10.1099/0022-1317-83-7-1799

[21] Hughes AL, Westover K, da Silva J, O'Connor DH, Watkins DI (2001) Simultaneous positive and purifying selection on overlapping reading frames of the tat and vpr genes of simian immunodeficiency virus. Journal of Virology 75: 7966–72. 11483741 10.1128/JVI.75.17.7966-7972.2001 PMC115040

[22] Hughes AL, Hughes MA (2005) Patterns of nucleotide difference in overlapping and non-overlapping reading frames of papillomavirus genomes. Virus Res 113: 81–88. 15913825 10.1016/j.virusres.2005.03.030

[23] Yockey HP (1992) Information Theory in Molecular Biology. Cambridge: Cambridge University Press.

[24] Yang Z (2006) Computational molecular evolution. Oxford: Oxford University Press.

[25] Shannon CE (1948) A mathematical theory of communication. Bell system technical journal 27.

[26] D'Argenio PR, Jeannet B, Jensen HE, Larsen KG (2001) Reachability analysis of probabilistic systems by successive refinements. In: APM-PROBMIV.

[27] Zhang Z, Li J, Yu J (2006) Computing ka and ks with a consideration of unequal transitional substitutions. BMC Evolutionary Biology 6: 44. 16740169 10.1186/1471-2148-6-44 PMC1552089

[28] Cover TM, Thomas JA (2006) Elements of Information Theory (Wiley Series in Telecommunications and Signal Processing). Wiley-Interscience.

[29] Schneider A, Cannarozzi G, Gonnet G (2005) Empirical codon substitution matrix. BMC Bioinformatics 6. 10.1186/1471-2105-6-134 PMC1173088 15927081

[30] Krakauer D (2000) Stability and Evolution of Overlapping Genes. Evolution; International Journal of Organic Evolution 54: 731–739. 10937248 10.1111/j.0014-3820.2000.tb00075.x

[31] Miyata T, Yasunaga T (1980) Molecular evolution of mRNA: a method for estimating evolutionary rates of synonymous and amino acid substitutions from homologous nucleotide sequences and its application. J Mol Evol 16: 23–36. 6449605 10.1007/BF01732067

[32] Kryazhimskiy S, Plotkin JB (2008) The population genetics of dN/dS. PLoS Genet 4: e1000304+. 10.1371/journal.pgen.1000304 PMC2596312 19081788

[33] Hein J, Stovlbaek J (1995) A maximum-likelihood approach to analyzing nonoverlapping and overlapping reading frames. Journal of Molecular Evolution 40: 181–9. 7699722 10.1007/BF00167112

[34] Pedersen AM, Jensen JL (2001) A dependent-rates model and an MCMC-based methodology for the maximum-likelihood analysis of sequences with overlapping reading frames. Mol Biol Evol 18: 763–76. 11319261 10.1093/oxfordjournals.molbev.a003859

[35] Fonseca M, Harris D, Posada D (2014) Origin and Length Distribution of Unidirectional Prokaryotic Overlapping Genes. G3 4: 19–27. 24192837 10.1534/g3.113.005652 PMC3887535

[36] Rogozin I, Spiridonov A, Sorokin A, Wolf Y, Jordan I, et al (2002) Purifying and directional selection in overlapping prokaryotic genes. Trends in Genetics 18: 228–232. 12047938 10.1016/s0168-9525(02)02649-5

[37] Fukuda Y, Washio T, Tomita M (1999) Comparative study of overlapping genes in the genomes of Mycoplasma genitalium and Mycoplasma pneumoniae. Nucleic Acids Research 27: 1847–1853+. 10101192 10.1093/nar/27.8.1847 PMC148392

[38] Fukuda Y, Nakayama Y, Tomita M (2003) On dynamics of overlapping genes in bacterial genomes. Gene 323: 181–187. 14659892 10.1016/j.gene.2003.09.021

[39] Cock P, Whitworth D (2007) Evolution of Gene Overlaps: Relative Reading Frame Bias in Prokaryotic Two-Component System Genes. Journal of Molecular Evolution 64: 457–462. 17479344 10.1007/s00239-006-0180-1

[40] Cock PJA, Whitworth DE (2010) Evolution of relative reading frame bias in unidirectional prokaryotic gene overlaps. Mol Biol Evol 27: 753–6. 20008458 10.1093/molbev/msp302

[41] Sabath N, Graur D, Landan G (2008) Same-strand overlapping genes in bacteria: compositional determinants of phase bias. Biology Direct 3: 36+. 18717987 10.1186/1745-6150-3-36 PMC2542354

[42] Haughton D, Balado F (2013) Biocode: Two biologically compatible algorithms for embedding data in non-coding and coding regions of dna. BMC Bioinformatics 14: 121+. 23570444 10.1186/1471-2105-14-121 PMC3698116

