# Supplementary material for: Selection Pressure in Alternative Reading Frames
Source: PLoS One. 2014 Oct 1;9(10):e108768. doi: 10.1371/journal.pone.0108768 (PMC4182739; doi:10.1371/journal.pone.0108768)
Supplement: File S1 — Additional Data and Figures. Contains further information to verify the model predictions by comparison with simulation, another method to determine the selection pressure, different investigations on the conditional entropy and mutual information as well as a method to show the robustness of results. (PDF) [file pone.0108768.s001.pdf]

SUPPORTING INFORMATION:

Selection Pressure in Alternative Reading Frames

Katharina Mir[1,†] and Steffen Schober[1],
1 Institute of Communications Engineering, Ulm University, Ulm, Germany
† Corresponding author: katharina.mir@uni-ulm.de

Model verification

In order to show that the approach presented in *Framework of Evolutionary Model* works in general, we reveal that the transition matrix P^f in the non-coding reading frames $f \in \{-1, \pm 2, \pm 3\}$ and the corresponding codon usage π^f matches simulation results. Therefore we consider in reading frame $+1$ a sequence X consisting of $n_G = 10^6$ independent and identical distributed (IID) random codons $c_1, c_2, \ldots, c_{n_G}$, where $c_j \in \mathcal{C}_{61}$ is drawn according to the codon usage $\pi^{+1}(c_j)$ of the original genome. X evolves to sequence Y according the evolutionary channel given in $+1$ with parameters $\kappa = 1.0$, $t = 1.0$ and $\omega = 0.3$. Given the sequences X and Y we are now able to determine the transition matrix observed in each frame P^f_{Sim} as well as the codon usage π^f_{Sim} per frame. From the codon usage, we immediately get the amino acid distribution.

A measure to compare two probability mass functions $p_X(x), q_X(x)$ $\forall x : p_X(x), q(x) > 0$, is the Kullback-Leibler divergence over all amino acids (plus stop) $\mathcal{A}^*$, e.g., [1]

$$D(P||Q) = \sum_{x \in \mathcal{A}^*} p_X(x) \log_2 \frac{p_X(x)}{q_X(x)}.$$

Further, we calculated the ℓ^2 norm (Euclidean distance) by

$$||P - Q||_2 = \sqrt{\sum_{x \in \mathcal{A}^*} (p_X(x) - q_X(x))^2}.$$

The distances of the amino acid distribution of the simulation compared with the calculations of the model are given in Table S1. Additionally, the amino acid distributions of the simulation against a random amino acid distributions (RND), where the probabilities of all amino acids are determined according to the GC content of the organism are given.

Table S1. Kullback-Leibler divergence and Euclidean distance of the amino acid distribution of the simulation with the calculations of the model.

Frame	$\|\|Sim - Model\|\|$	$D(Sim\|\|Model)$
$+1$	$7.6169 \cdot 10^{-4}$	$1.5623 \cdot 10^{-5}$
$+2$	$9.0041 \cdot 10^{-4}$	$2.7991 \cdot 10^{-5}$
$+3$	$1.2633 \cdot 10^{-3}$	$3.8891 \cdot 10^{-5}$
-1	$8.0160 \cdot 10^{-4}$	$2.1625 \cdot 10^{-5}$
-2	$8.4170 \cdot 10^{-4}$	$2.1848 \cdot 10^{-5}$
-3	$1.0425 \cdot 10^{-3}$	$2.6290 \cdot 10^{-5}$
RND	$8.1741 \cdot 10^{-2}$	$1.9047 \cdot 10^{-1}$

Selection pressure

As the comparison of synonymous and nonsynonymous substitution rates is used to quantify the natural selection on proteins [2], several methods have been developed to determine ω, either by Maximum Likelihood estimation [3] or by heuristic counting methods introduced in [4, 5]. For the counting method, there have been numerous improvements published e.g., [2, 6–11]. Unfortunately some methods produce different estimation results, as they are not very robust towards the model assumptions. Typically, the methods overestimate the number of synonymous substitutions and underestimate the number of nonsynonymous substitutions [11].

We apply the method of Nei and Gojobori (NG) [6] to estimate the synonymous and nonsynonymous rate ratio. For the model, we determined the joint probability that codon c_x evolved to codon c_y from the stationary distribution and the transition matrix. Figures S1 and S2 show that our prediction models exactly the behaviour of the simulated sequences. Note, the red line (estimation of frame +1 should be linear with slope one. This deviation is due to a bias in the NG method, which is described, e.g. in [11].

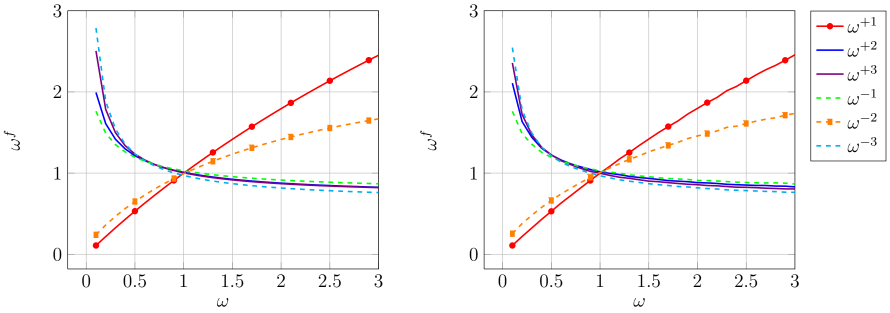

(a) Model (b) Simulation

Figure S1. Selection pressure estimated with NG method of simulation and model prediction for $\kappa = 1.0$, $t = 1.0$

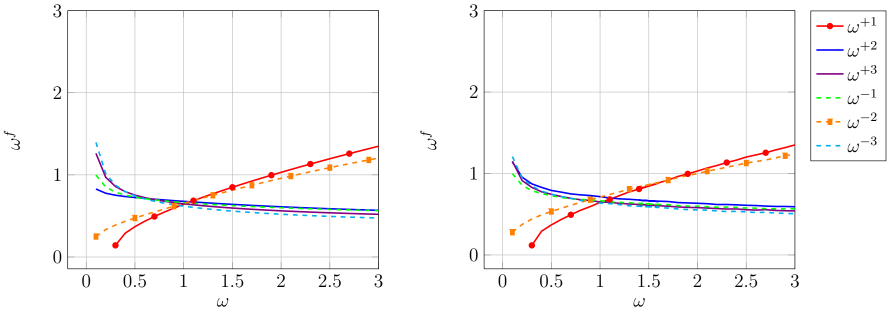

(a) Model (b) Simulation

Figure S2. Selection pressure estimated with NG method of simulation and model prediction for $\kappa = 5.0$, $t = 5.0$

The selection pressure determined from the rate matrix Q is presented in Figure S3. A comparison with the predictions of the NG method in Figures S1 and S2 shows the same tendencies, but the method calculating the rate ratio using the rate matrix is robust over different values of ω.

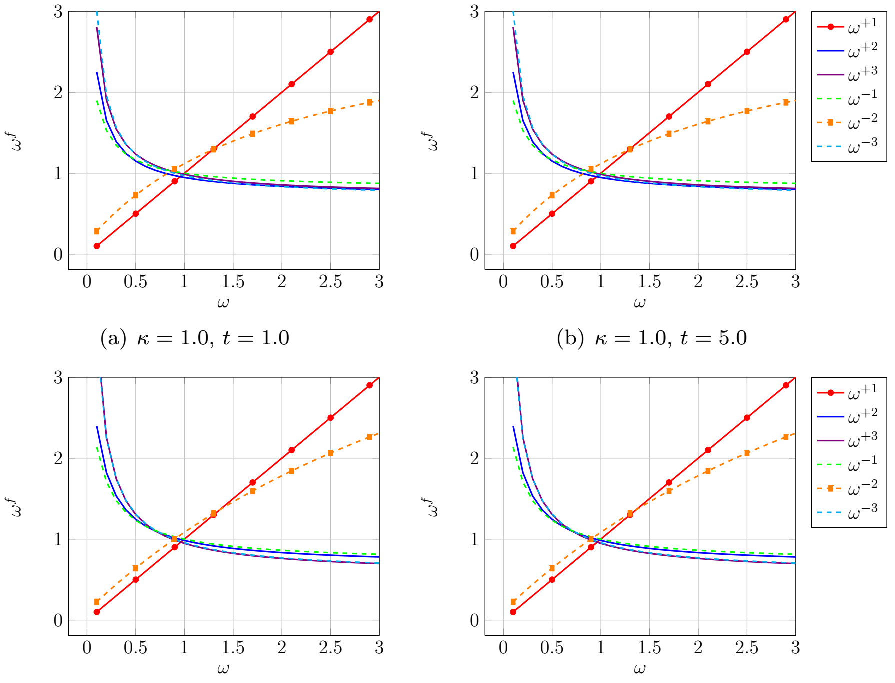

(a) $\kappa = 1.0$, $t = 1.0$

(b) $\kappa = 1.0$, $t = 5.0$

(c) $\kappa = 5.0$, $t = 1.0$

(d) $\kappa = 5.0$, $t = 5.0$

Figure S3. Selection pressure from modified rate matrix Q^f

Conditional entropy and mutual information

The conditional entropy and mutual information for different values of ω is shown in Figure S4.

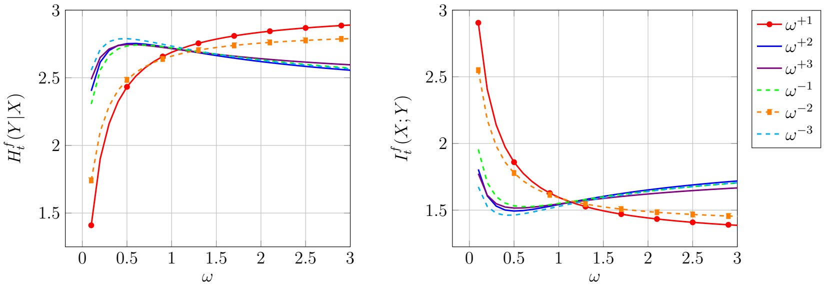

(a) Conditional entropy (b) Mutual information

Figure S4. Estimation of conditional entropy and mutual information for $\kappa = 1.0$, $t = 1.0$

Robustness of results

Given the evolution matrix in the coding reading frame $P^{+1}_{Y|X}$ and some stationary distribution π^{+1}, the assumption of independent and identical distributed codons can be applied to determine transition matrix in different reading frames. Here is the example of frame +2

$$P^{+2}_{Y|X}(y_2^1 y_3^1 y_1^2 | x_2^1 x_3^1 x_1^2) = \frac{\sum_{x_1^1 \in \mathcal{N}} \sum_{y_1^1 \in \mathcal{N}} \pi^{+1}(x_1^1 x_2^1 x_3^1) \cdot P^{+1}(y_1^1 y_2^1 y_3^1 | x_1^1 x_2^1 x_3^1)}{\sum_{x_1^1 \in \mathcal{N}} \pi^{+1}(x_1^1 x_2^1 x_3^1)} \cdot \frac{\sum_{x_2^2, x_3^2 \in \mathcal{N}} \sum_{y_2^2, y_3^2 \in \mathcal{N}} \pi^{+1}(x_1^2 x_2^2 x_3^2) \cdot P^{+1}(y_2^1 y_2^2 y_3^2 | x_2^1 x_2^2 x_3^2)}{\sum_{x_2^2, x_3^2 \in \mathcal{N}} \pi^{+1}(x_1^2 x_2^2 x_3^2)}.$$

Given the transition matrix $P^f_{Y|X}$ of a Markov chain the corresponding stationary distribution π^f in each reading frame can be easily determined.

References

1. Cover TM, Thomas JA (2006) Elements of Information Theory (Wiley Series in Telecommunications and Signal Processing). Wiley-Interscience.

2. Yang Z, Nielsen R (2000) Estimating synonymous and nonsynonymous substitution rates under realistic evolutionary models. Molecular Biology and Evolution 17: 32–43.

3. Goldman N, Yang Z (1994) A codon-based model of nucleotide substitution for protein-coding DNA sequences. Molecular Biology and Evolution 11: 725-736.

4. Miyata T, Yasunaga T (1981) Molecular evolution of mRNA : A method for estimating evolutionary rates of synonymous and amino acid substitutions from homologous nucleotide sequences and its application. Genetics 98: 641-657.

5. Perler F, Efstratiadis A, Lomedico P, Gilbert W, Kolodner R, et al. (1980) The evolution of genes: the chicken preproinsulin gene. Cell 20: 555 - 566.

6. Nei M, Gojobori T (1986) Simple methods for estimating the numbers of synonymous and nonsynonymous nucleotide substitutions. Molecular biology and evolution 3: 418–426.

7. Li WH, Wu CI, Luo CC (1985) A new method for estimating synonymous and non-synonymous rates of nucleotide substitutions considering the relative likelihood of nucleotide and codon changes. Molecular Biology and Evolution 2: 150-174.

8. Li WH (1993) Unbiased estimation of the rates of synonimous and nonsynonimous substitution. Journal of Molecular Evolution 36: 96-99.

9. Pamilo P, Bianchi NO (1993) Evolution of the Zfx and Zfy genes: rates and interdependence between the genes. Mol Biol Evol 10: 271–81.

10. Comeron JM (1995) A method for estimating the numbers of synonymous and nonsynonymous substitutions per site. J Mol Evol 41: 1152–9.

11. Ina Y (1995) New methods for estimating the numbers of synonymous and nonsynonymous substitutions. J Mol Evol 40: 190–226.
